# High-field modulated ion-selective field-effect-transistor (FET) sensors with sensitivity higher than the ideal Nernst sensitivity

**DOI:** 10.1038/s41598-018-26792-9

**Published:** 2018-05-29

**Authors:** Yi-Ting Chen, Indu Sarangadharan, Revathi Sukesan, Ching-Yen Hseih, Geng-Yen Lee, Jen-Inn Chyi, Yu-Lin Wang

**Affiliations:** 1 Institute of Nanoengineering and Microsystems, National Tsing Hua University, Hsinchu, 300 Taiwan Republic of China; 2 Department of Power Mechanical Engineering, National Tsing Hua University, Hsinchu, 300 Taiwan Republic of China; 3 Department of Electrical engineering, National Central University, Jhongli City, Taoyuan County, 320 Taiwan Republic of China

## Abstract

Lead ion selective membrane (Pb-ISM) coated AlGaN/GaN high electron mobility transistors (HEMT) was used to demonstrate a whole new methodology for ion-selective FET sensors, which can create ultra-high sensitivity (−36 mV/log [Pb^2+^]) surpassing the limit of ideal sensitivity (−29.58 mV/log [Pb^2+^]) in a typical Nernst equation for lead ion. The largely improved sensitivity has tremendously reduced the detection limit (10^−10^ M) for several orders of magnitude of lead ion concentration compared to typical ion-selective electrode (ISE) (10^−7^ M). The high sensitivity was obtained by creating a strong filed between the gate electrode and the HEMT channel. Systematical investigation was done by measuring different design of the sensor and gate bias, indicating ultra-high sensitivity and ultra-low detection limit obtained only in sufficiently strong field. Theoretical study in the sensitivity consistently agrees with the experimental finding and predicts the maximum and minimum sensitivity. The detection limit of our sensor is comparable to that of Inductively-Coupled-Plasma Mass Spectrum (ICP-MS), which also has detection limit near 10^−10^ M.

## Introduction

Heavy metals have been used in various applications from ancient times and although the health hazards posed by the heavy metals are known to mankind, they still find their way to our bodies by contamination of natural systems and continued exposure to products that contain heavy metals. Although the term “Heavy metal” doesn’t have consistent definition, it is widely used to represent a group of metals and metalloids that have been related to environment pollution, toxicity or bio-toxicity^1^. Unlike other toxins, it is impossible for liver to metabolize or decompose heavy metals. It is hence easy for accumulation of heavy metals in brain, kidney and other organs, which leads to life threatening health conditions. The most commonly seen heavy metals such as lead, mercury, cadmium and arsenic are all listed in the top 10 chemicals of major public concern by the World Health Organization in 2017. Others like manganese, nickel, zinc, copper, et cetera are also harmful for human body^2–4^.

Lead is one of the most widely occurring heavy metal in our day to day life since it exists in polluted air, hair dye, paint, drinking water, food, fertilizer etc.^5–7^. Lead is hazardous, especially due to its irrevocable damage to the nervous system of children, which can lead to blood related disorders and encephalopathy^8^. Apart from children, long-term exposure in adults to lead and its salts (especially soluble and strongly oxidized PbO_2_) may cause kidney damage and colic abdominal pain^9,10^. Factory emissions containing lead pollutes groundwater and soil, thus entering the natural ecosystem. As humans ingest lead-contaminated foods, it will accumulate in bones as excessive levels of Pb which is larger than the natural metabolizing rate of 300 μg per day^11,12^. Thus, monitoring Pb concentration in water sources is imperative to minimize health hazards.

Several types of heavy metal ion detection methods have already been well developed including atomic absorption spectroscopy (AAS), x-ray fluorescence (XRF), inductively coupled plasma-atomic emission spectroscopy (ICP-AES), electrochemical and fluorescence techniques^13^. These laboratory based bench-top instruments can detect very low ion concentrations approaching to parts per trillion (1 ppt = 0.001 μg/L). Even though very low detection limits can be achieved, the laboratory based techniques are highly inconvenient and quite expensive for rapid and dynamic screening of contaminants. Apart from traditional laboratory-based detection methods, the ion-selective electrode (ISE) based on polymeric membranes containing ionophores as acceptors for target ions is widely recognized for its numerous advantages such as easy fabrication, short response time and high selectivity. These features are very useful in clinical analyses, rapid chemical detection and environmental monitoring. The sensing mechanism of ISE is described by Nernst equation and site binding theory. The ideal Nernst sensitivity for divalent heavy metal ion such as Pb is 29.58 mV/decade concentration of ion. This sensor response however, is inadequate to detect trace amounts of contaminants in sources such as drinking water that are indispensable part of human daily life. Also, the operation of ISE is not easy, and it cannot achieve appreciable detection limit for Pb. It is usually acceptable for a traditional Pb ISE to have a detection limit of 10^−7^ M^11,13,14^, while government regulations for Pb in tap water and potable water are below 10^−7^ M^2^. Exposure to heavy metals especially lead continues to be prevalent and has increased rapidly in some areas^5^. The recent electrochemical methods such as photoelectrochemical sensing^15–20^ and optical methods such as fluorescence, SERS and FRET sensors^21,22^ for heavy metal ion detection have achieved very low detection limits and potentially provides opportunities for compact systems. However, they require labels and other specific reagents, and dyes are susceptible to photobleaching. The use of molecular probes and specific reagents such as nucleotides as receptors will potentially limit the test environment (pH, ionic strength). Electrochemical sensing requires charge transfer reactions leading to redox currents and optical sensors require light source for excitation. These methods are generally more complex owing to their test requirements and signal transduction mechanism.

We need to develop a heavy metal screening method that can be directly employed in drinking water and tap water sources to dynamically monitor the variation of lead ion concentration. Such a device needs to have very simple operation steps and achieve at least two orders lower detection limit compared to the legal limit for lead. The sensor technology presented in this work is a purely electronic, high field modulated field effect transistor (FET) sensor that can directly detect heavy metal ions in solution with a dynamic range of 10^−10^ M to 10^−5^ M, which is ideal for water quality monitoring in every day scenarios such as drinking water and tap water. In this research, we have utilized the ion selective membrane (ISM) in combination with a high sensitivity electrical double layer (EDL) gated field effect transistor (FET) to overcome the drawbacks of the traditional methods and improve the sensing characteristics and device applicability. FET has several advantages such as rapid measurement, easy signal read out and high sensitivity. Among various types of FET, we chose to employ AlGaN/GaN high electron mobility transistor (HEMT) for transduction due to numerous advantages such as chemical and physical perseverance, speedy operation and high sensitivity to surface charges^23^. In our previous works, we developed a highly sensitive EDL gated FET biosensors intended for rapid screening of proteins with ease and convenience, at a very low cost^24^. By combining the EDL FET sensor with the traditional ion selective membrane (ISM), we developed a technique that can provide a high sensitivity, low detection limit and rapid heavy metal ion screening in water sources to monitor heavy metals. The advantages and the features of our ISM based EDL gated FET sensor include, (1) high sensitivity heavy metal ion detection that surpasses the ideal Nernstian response by large, (2) very low detection limit for Pb (10^−10^ M) comparable to the conventional bench top instruments, (3) speedy detection in 10 mins, (4) steady baseline and repeatability, due to short duration single pulse bias voltage, which generates less heat and thermal noise, leading to less drifting and variations, (5) extremely low cost, (6) miniaturized device configuration, enabling multi-purpose device applicability, (7) ease and convenience of operation, which enables consumers to rapidly screen for heavy metal ions without any pre-training.

In the present work, we have developed a semi-empirical quantitative model for our unique Pb-ISHEMT (lead-ion selective high electron mobility transistor) sensors and proved ultra-high sensitivity beyond the ideal sensitivity in typical Nernst equation by choosing suitable designs and bias conditions. When the distance between the gate electrode and the HEMT channel is reduced, it can provide high electric field in the test solution to modulate the formation of EDL on the ISHEMT channel. Thus, when target heavy metal ions bind to the ISM, the transistor current gain changes, with very high sensitivity which is appreciably higher than the ideal Nernstian slope. The semi-empirical model developed herein describes the dependence of sensitivity on applied gate bias and distance between the gate electrode and ISHEMT channel. We also discuss how the gap between the gate electrode and the HEMT channel affects the sensitivity. The prediction of the sensitivity is consistent with the results. This model consistently proves that the traditional ISE or ISMFET works in a large distance between the gate electrode and transistor channel, resulting in low electric field operation and the constraints of ideal Nernst response. This model is applicable to all types of ISMFET sensor which further improves the future prospects of this methodology to be used for the detection of multitudes of targets in water sources (tap and drinking water), foods, beverages and natural systems such as rivers and lakes.

## Results

**Figure 1:**
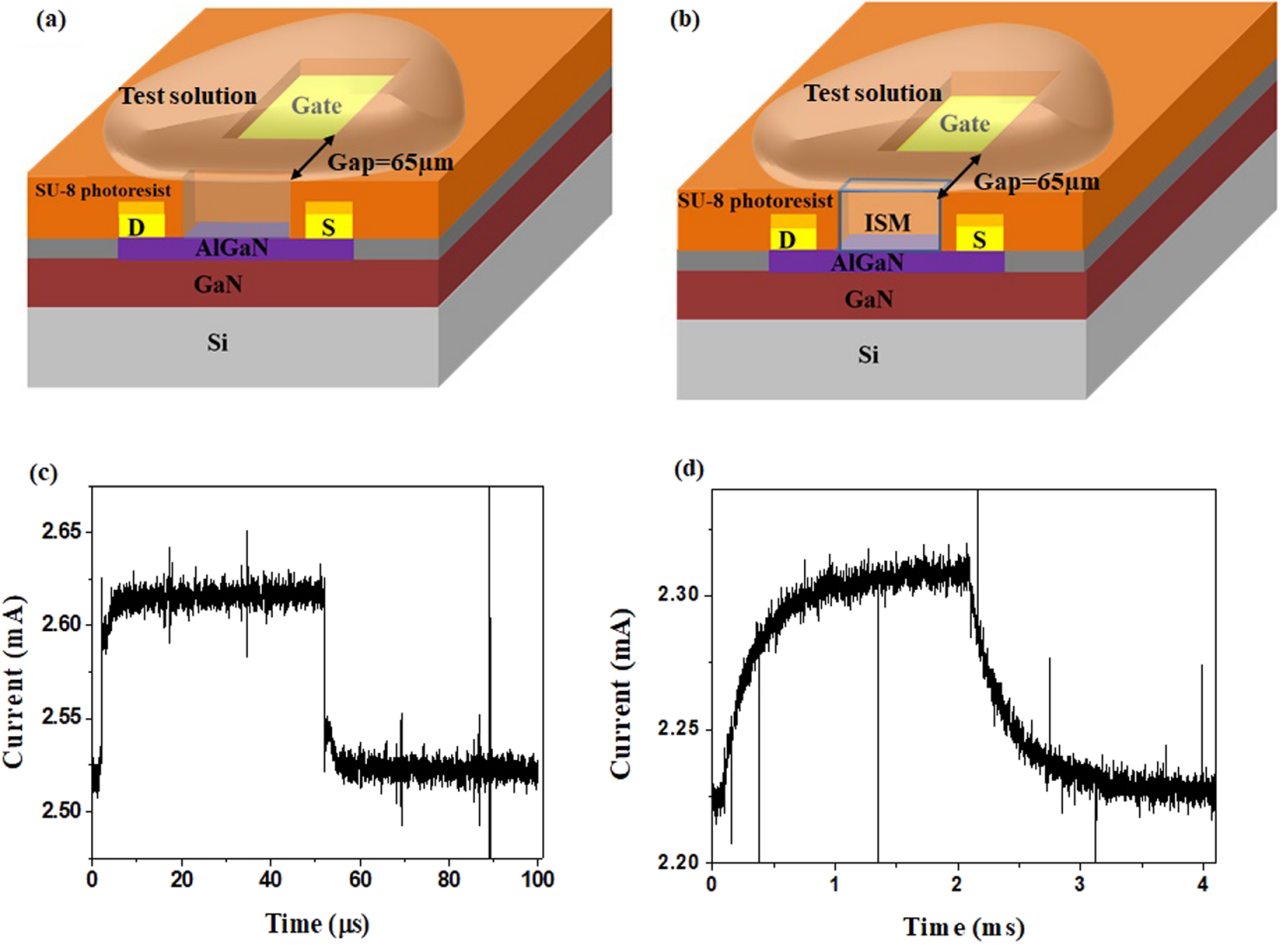
High field gated AlGaN/GaN HEMT sensor. Schematic representation of AlGaN/GaN HEMT sensor (**a**) without ISM (**b**) with ISM. Electrical characteristics of AlGaN/GaN HEMT sensor (**c**) without ISM (**d**) with ISM.

The schematic structure of Pb-ISHEMT is shown in Fig. 1. The AlGaN/GaN epiwafer undergoes ICP etching to define the device active area followed by ohmic contact deposition, metal interconnection and gate electrode deposition. The whole device is passivated and gate electrode and HEMT channel are selectively opened using photolithography. The opening on gate reference electrode is positioned at 65 µm away from the HEMT channel opening, on the same plane (Fig. 1(a)). The transistor active channel is formed by the high density of electron concentration at the AlGaN and GaN interface, which leads to very high conductivity. Thus, we can ignore the potential drop in the FET channel. The test solution comes in contact with the openings on the gate electrode and the transistor channel, essentially acting as the liquid dielectric between two metal plates. The lead ISM is coated on the channel opening, without coming in contact with the gate electrode, as shown in Fig. 1(b). The electrical characteristics of Pb-ISHEMT are shown in Fig. 1(c,d). Without ISM, when pulsed gate voltage is applied, mobile ions in the test solution accumulate at the gate electrode and transistor channel interfaces, forming EDL on both sides, setting up a solution capacitance C_s_. It can modulate the transistor dielectric capacitance C_d_, whereby changing the drain current. The structure and mechanism of this ion-gated liquid FET has been investigated in detail in our previous works^24^. The drain current response of bare AlGaN/GaN HEMT is depicted in Fig. 1(c). It is important to note the definition of sensor signal which is the current gain. Current gain or simply ‘gain’ of our sensor is defined as the change in transistor drain current before and after gate bias is applied. It is not exact but analogous to the transconductance gain defined in the context of traditional FET model. This sensor index is used as the signal in order to avoid using the absolute drain current value of HEMT, which is prone to variations owing to thermal drift and random noise. Therefore current gain offers better stability and a steady baseline for sensor measurement. When ISM is coated on the HEMT channel, the electrical characteristics are altered (as in Fig. 1(d)). The ISM is a polymer matrix of organic charged molecules that are mobile under an electric field. When gate pulse is applied, the EDL is formed at the gate electrode-solution interface and within the ISM which modulates C_d_ and hence the transistor drain current, as shown in Fig. 1(d). The relaxation time of drain current is longer in device with ISM (~1 ms) compared to device without ISM (~15 µs) because of the denser polymer gel like matrix and the lesser mobility of organic charged molecules forming the EDL. When lead ions in the test solution are bound to the ISM, C_d_ will be altered, and the drain current response will be proportional to the ion concentration, thereby providing a sensitive and quantitative determination of the lead concentration in the test sample.

**Figure 2:**
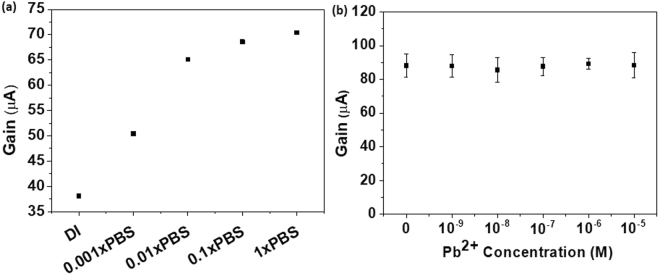
Characteristics of AlGaN/GaN HEMT without ISM. (**a**) Current gain response of sensor with increasing ionic strength of test solution. (**b**) Current gain response of sensor for increasing concentration of lead.

Figure 2 describes the current response of bare AlGaN/GaN HEMT in different test solutions. When ion concentration of the test solution is varied, the current gain of bare HEMT varies accordingly, as shown in Fig. 2(a). In DI water, with extremely low mobile ions, the device gain is considerably small. This is due to the lack of mobile ions to contribute to the formation of solution capacitance C_s_ to modulate the drain current. When ion concentration in the solution is altered by increasing the ion concentration from 0.001X to 1X PBS, the current gain of the bare HEMT increases as the ion concentration increases. This shows working principle of the EDL gated HEMT sensor. However, when different concentrations of lead ions are introduced to the bare HEMT device, the current gain does not change appreciably, as shown in Fig. 2(b). This is because the ionic strength of the test solution is not varied considerably when lead ions are added, as the salt concentration of 0.02× PBS containing about 2 mM NaCl predominates the current response. Thus bare AlGaN/GaN HEMT does not respond to the lead ions present in the solution and a specific receptor is required to capture the lead ions such as ISM, in order to function as Pb-ISHEMT. The results on Fig. 2(b) also show that the heavy metal ions present in the test solution which are not specifically captured on the device, do not contribute to the drain current response of the device. This further improves the selectivity of the sensor towards the target ion.

**Figure 3:**
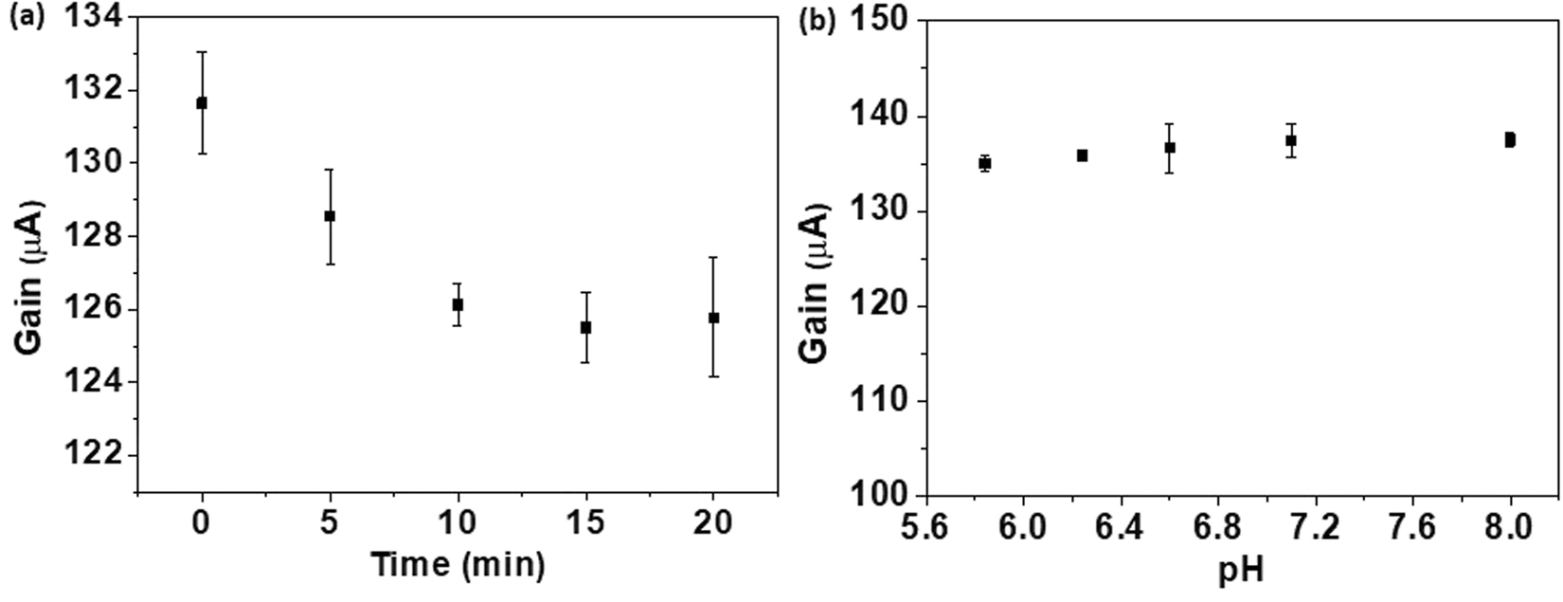
Characteristics of Lead ion selective HEMT (Pb-ISHEMT). (**a**) Current gain response of Pb-ISHEMT through time. (**b**) Current gain response of Pb-ISHEMT for varying pH of test solution.

The device characteristics with ISM embedded on the HEMT channel region is depicted in Fig. 3. As the device with ISM containing Pb ionophore comes in contact with solution and gate pulse is applied, the current gain takes some time to reach a steady baseline, as shown in Fig. 3(a). This can be explained as the transition of Pb-ISHEMT sensor from air to liquid interface and proper device wetting by the test solution. The dynamic current gain response achieves steady state after 10–15 minutes of contact with the test solution, which is the response time for the Pb-ISHEMT sensor. Since the lead ion sensor is intended to be used for real time water quality monitoring, it is worthwhile to investigate the pH dependence of the sensor. In Fig. 3(b), the current gain of Pb-ISHEMT does not significantly change as the pH of the test solution is varied from 5.6 to 8. This shows that the sensor operation is stable in the pH range 5–8. This is quite convenient as the pH of drinking water or tap water is closely maintained in this pH range. The typical transfer characteristic of the Pb-ISHEMTs and the current gain measured at time domain as pulse signals are shown in supplementary information as Figs S1 and S2, respectively.

**Figure 4:**
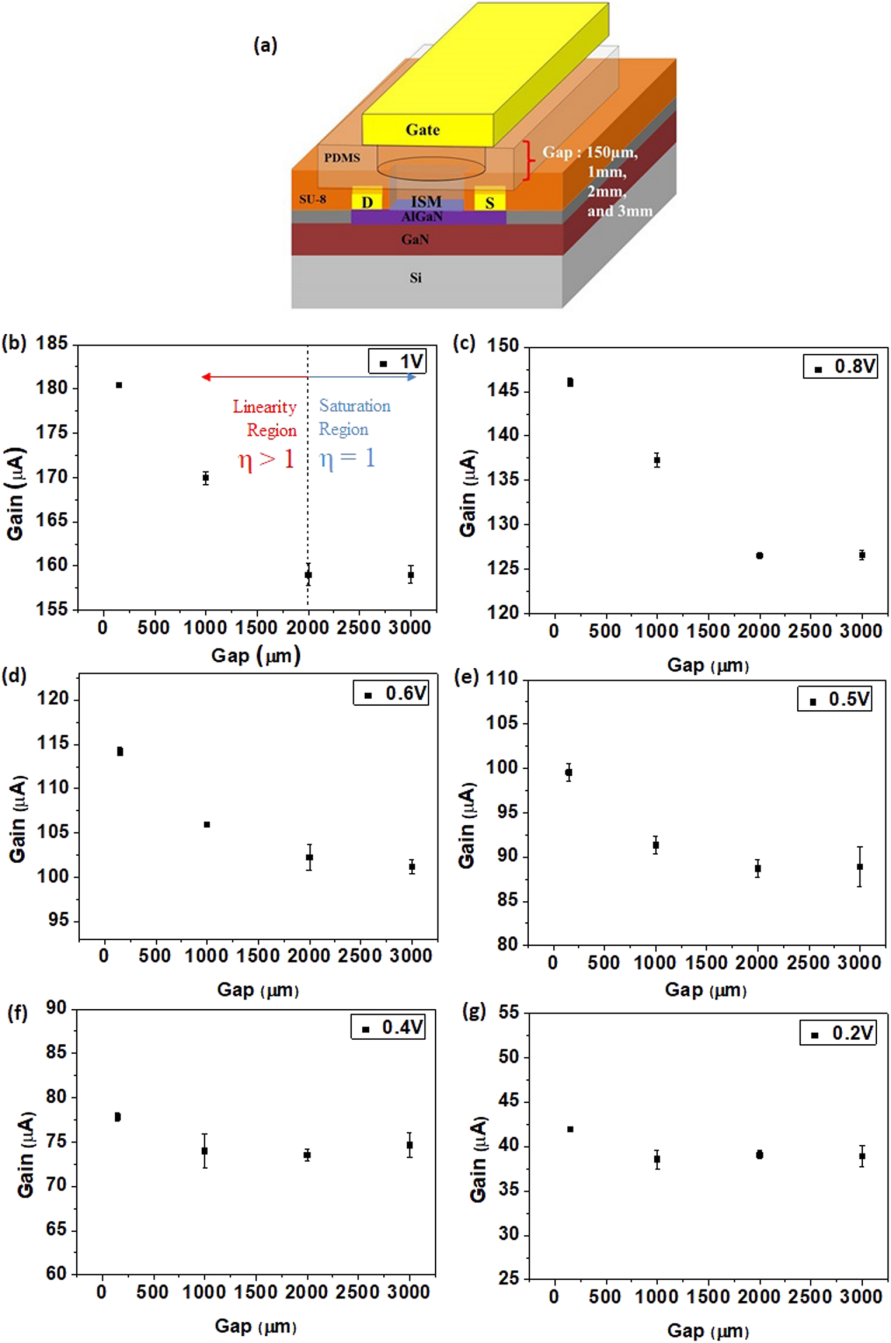
Effect of gate electrode gap and applied V_g_ on current gain. (**a**) Test setup for evaluating the sensor response for different applied electric field. (**b**–**g**) Current gain versus gap for fixed V_g_.

The working mechanism of Pb-ISHEMT can be better understood by examining the gating mechanism of the sensor. Figure 4 shows the current gain response of Pb-ISHEMT under different device configurations of varying gaps between the gate electrode and ISHEMT channel. The gap between the gate electrode and the channel is changed in vertical direction using a PDMS spacer, as depicted in Fig. 4(a). The in-plane gate electrode in the typical Pb-ISHEMT is not used to apply the gate bias voltage. Instead, a vertical gold gate electrode is placed on top of the PDMS spacer with varying thickness to modulate the gap between the gate electrode and ISHEMT channel. In this way, the electric field strength of Pb-ISHEMT is varied and the sensor response can be investigated. The PDMS spacer has a drill hole at the center to form a well which is then filled with 0.02X PBS such that the vertical gate electrode and ISHEMT channel come in contact via the solution in the PDMS spacer well. The range of thickness of the PDMS spacer was from 150 µm to 3 mm. With different gaps between the gate electrode and the ISHEMT channel, we obtain different current gain values as shown in Fig. 4. Corresponding to each gap between gate electrode and ISHEMT channel, we tested the current gain with different gate pulse voltage V_g_ ranging from 0.2 V to 1 V (Fig. 4(b–g)). The current gain decreases as the vertical gap increases till it becomes stable after a certain gap. For example, in Fig. 4(b), when a gate voltage of 1 V is applied, the current gain shows considerable gap dependence along a decreasing trend before reaching saturation around a gap of 2 mm. Thus we can obtain two different gap dependent current responses. When the gap is sufficiently small, i.e, less than 2 mm, the gain varies linearly with respect to the gap. This can be called as the linear or high field region. When the gap is larger than 2 mm, the current gain of the sensor is independent of the gap and the applied V_g_. This can be called as the saturation or low field region where the electric field is constant and approaches to that of the bulk solution. In Fig. 4(b) through (g), the applied gate bias is varied from 1 V to 0.2 V, for different gaps of 150, 1000, 2000 and 3000 µm. We can clearly see that for lower gate voltages, the saturation of current gain is achieved at a smaller gap, i.e., around 1 mm. Thus, the current gain is dependent on the applied gate voltage and the gap between the gate electrode and the ISHEMT channel. This means that when we operate the sensor under the linear region, Pb-ISHEMT can be modulated by the applied electric field and larger current gain can be obtained.

**Figure 5:**
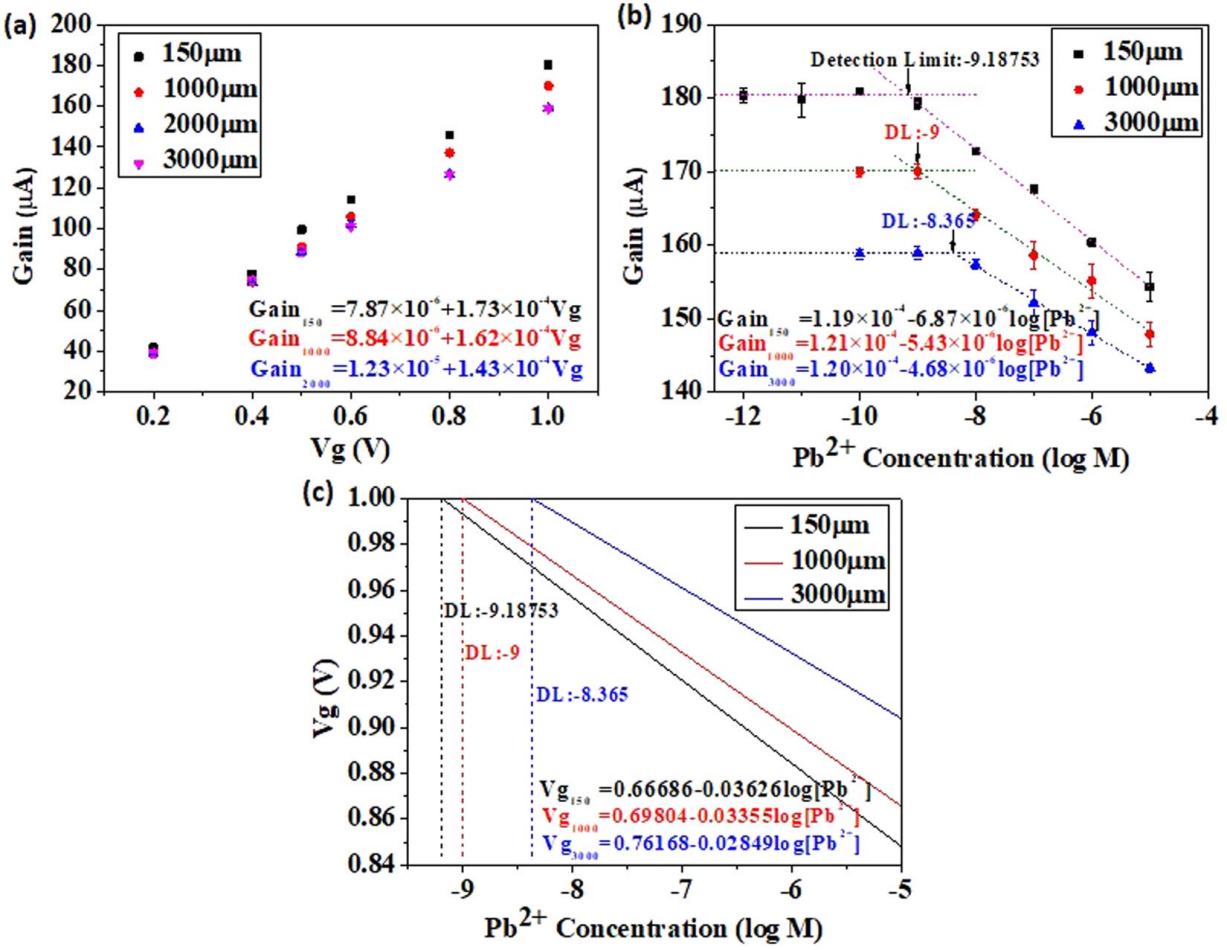
Lead ion detection using Pb-ISHEMT and comparison of sensitivity. (**a**) Current gain response for different applied V_g_. (**b**) Current gain response for different lead ion concentration. (**c**) Effective V_g_ applied for different lead ion concentration.

It is intuitive to compare the current gain response of Pb-ISHEMT as a function of the applied gate voltage V_g_, for different gaps between gate electrode and ISHEMT channel, as shown in Fig. 5(a). Evidently, the current response of sensor or slope of the gain versus V_g_ graph at gaps above 2 mm (saturation region of operation) is nearly constant while the slopes under linear region of operation (150 µm to 1 mm) increase with decreasing gap. This means that the sensitivity of Pb-ISHEMT can be enhanced by employing the sensor to operate under linear region. The sensitivity of Pb-ISHEMT operated under the saturation region is clearly lower and independent of the electric field strength in the solution. In traditional ISE or ISM-FET the reference electrode is maintained at very large distances away from the FET^25–34^. Also, the reference electrode is mostly grounded or applied with a small bias such that the electric field strength between the reference electrode and the FET is extremely low. Therefore in the conventional methods, ISE or ISM-FET is operated in the saturation region, where the sensitivity lower and independent of the applied electric field. In this way, we can qualitatively compare the sensitivity of our Pb-ISHEMT and the traditional methods and develop a method to enhance the sensitivity even further to improve the detection limit of lead in the test solution.

A more comprehensive understanding of the sensing mechanism of Pb-ISHEMT can be obtained by quantitatively comparing the sensitivities of device operation in linear and saturation regions. Figure 5(b) shows the current gain change corresponding to different concentrations of lead ion in 0.02 × PBS, for gaps of 150 µm, 1 mm and 3 mm at 1 V V_g_. The sensitivity of Pb-ISHEMT at 150 µm and 1 mm gap (in linear region) is significantly higher than at 3 mm gap (saturation region) and the limit of detection is improved by one order in linear region of operation. This elucidates the significant advantage of our Pb-ISHEMT sensor which works in the linear of high field region of operation, in detecting trace amount of lead ion contamination. In order to carry out a quantitative comparison with the traditional methods, we need to consider Nernst equation that describes the response of ISE or ISM-FET:

1 $${E}_{ISM}=c+\frac{RT}{nF}\,\mathrm{log}\,A$$

where A represents the target ion concentration, R, T and F denote gas constant, temperature and Faraday’s constant respectively and n denotes the valence of the target ion.

In the case of lead ion detection, n = 2 and the ideal Nernst response is given by the equation:

2 $${E}_{ISM,ideal}=c+0.02958\,\mathrm{log}[P{b}^{2+}]$$

Thus the ideal Nernstian sensitivity would be −29.58 mV/decade lead ion concentration. While using ISE or ISM-FET several research groups have demonstrated sensors with near ideal Nernstian response and discussed methods to improve the sensitivity to approach ideal Nernst slope^34–44^. These improvements are primarily focused on the site binding theory by which enhancing the surface porosity or roughness of ISM provides more binding sites for target ions which brings the sensitivity closer to the Nernst response. However, some research also reports higher than Nernst response^27–33^, but without systematic analysis or quantitative model to validate the claim of enhanced sensitivity. In Fig. 5(c), we have derived an equation to obtain sensor calibration curve in terms of change in effective V_g_. The sensor response in gain versus V_g_ (Fig. 5(a)) and gain versus [Pb^2+^] (Fig. 5(b)) graphs can be combined to mathematically obtain the sensor response in the form of Vg expressed as a function of [Pb^2+^] (Fig. 5(c)). The sensor response at different gaps of 150 µm, 1 mm and 3 mm are fitted mathematically to generate their respective slopes which represent the sensitivity. When device is operated in saturation region (gap = 3 mm) the slope is −28.49 mV/decade [Pb^2+^] which is very close to the ideal Nernst slope. When the device is operated in linear or high field region (gap = 150 µm or 1 mm), the slopes are −36.26 and −35.67 mV/decade [Pb^2+^] which is considerably higher than the ideal Nernst sensitivity. Thus, we have demonstrated that traditional ISE or ISM-FET devices are operating in the saturation region which is limited by the ideal Nernst response. On the other hand, the gating mechanism used in our Pb-ISHEMT can provide sensitivity beyond the ideal Nernst response and obtain better limit of detection. This is due to the device operation in linear region, where the current gain of the sensor is dependent on the applied gate electrode voltage V_g_ and the gap between the gate electrode and the ISHEMT channel. We can then derive a semi-empirical model to incorporate the strong dependence of sensor response on the electric field strength in the solution as:

3 $${E}_{Pb-ISHEMT}=c+0.02958\eta \,\mathrm{log}[P{b}^{2+}]$$

where η is dependent on the applied V_g_ and the gap between the gate electrode and ISHEMT channel. In linear or high field region, η is greater than 1 and in saturation region η is equal to 1. In this way, we can quantitatively describe the sensing mechanism of Pb-ISHEMT and explain the ideal Nernst slope limited response of traditional ISE or ISM-FET.

## Discussion

**Figure 6:**
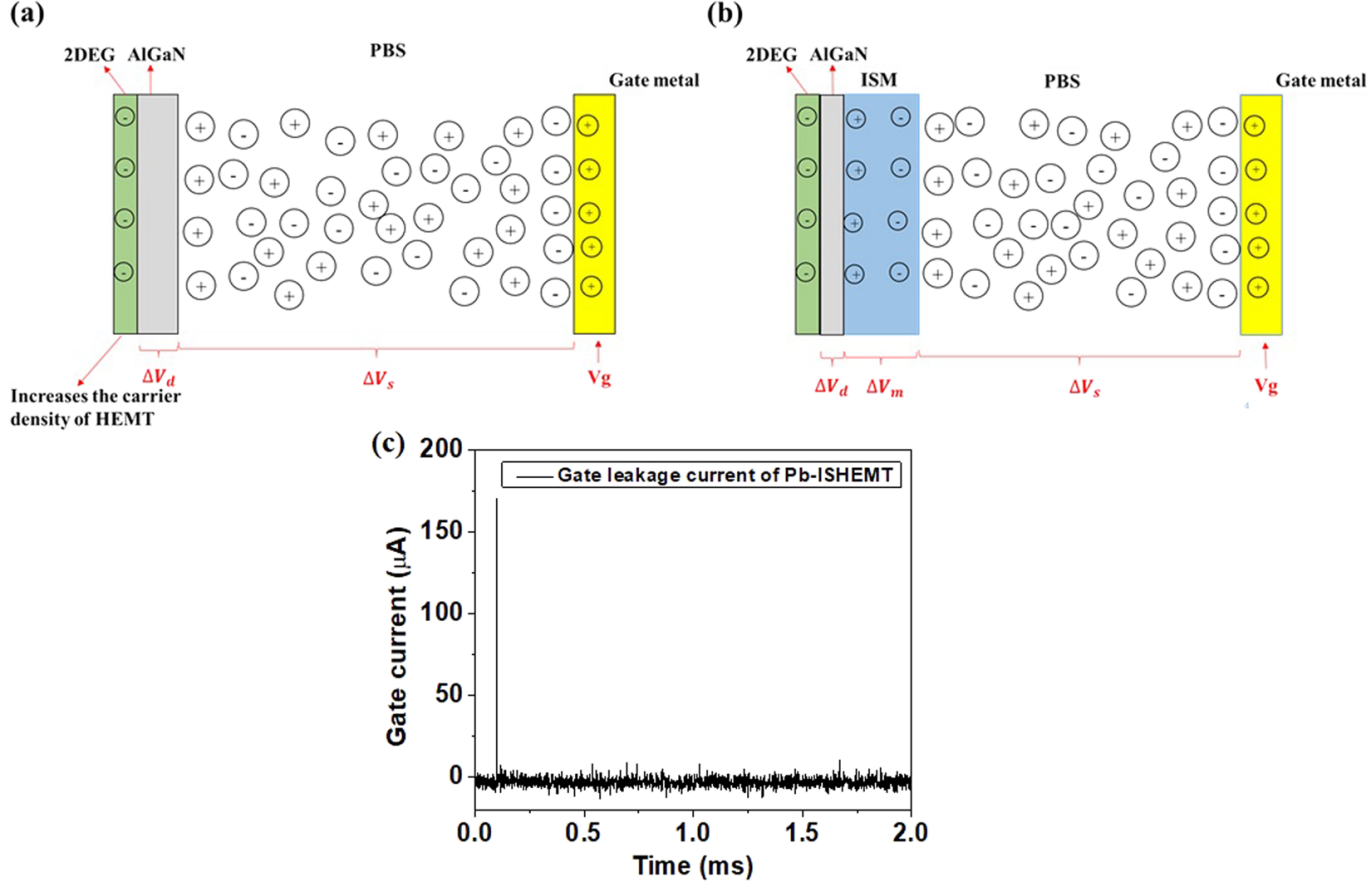
Characteristics of electrical double layer with and without ISM. Charge distribution in AlGaN/GaN HEMT sensor (**a**) without ISM (**b**) with ISM (**c**) Gate electrode leakage current of Pb-ISHEMT.

As previously discussed, traditional ISE or ISM-FET operates in the saturation region where the reference electrode and channel are far away such that electric field gradient in the solution is that of bulk of the solution. However, in our Pb-ISHEMT, electric field gradient exists in the solution between the gate electrode and the ISHEMT channel, due to the applied gate bias and the short gap between the two. We can compare the biasing conditions of our sensor to that of electrophoresis, in which an electric field is applied between two electrodes that is at least larger than 1 V/cm^45–47^. In our sensor, if we consider the typical gap of 150 µm and the applied V_g_ of 1 V, the field we apply across the solution is around ~67 V/cm. If we consider 1 V/cm as the critical field, our sensor works under high field operation where the applied electric field can modulate the sensor signal. It is intuitive to find out how high field modulation affects the sensor characteristics such as sensitivity. We can model our sensor as a two-plate capacitor, with dielectric medium in between. Figure 6(a,b) presents a schematic representation of the formation of electrical double layer at the gate electrode and channel interfaces, in the device without ISM and with ISM, respectively. In conventional ion selective electrode model, faradaic current or redox current is considered when high bias voltages are used. However, in this sensor design, we measure non-faradaic response, and this is verified by measuring the gate leakage current of HEMT, as shown in Fig. 6(c). The leakage current quickly reduces to almost zero under bias application which indicates that faradaic reactions do not occur at the solid/liquid interface. Thus, we assume a purely capacitive model and does not include the resistive component of overall impedance. If we apply a positive voltage on gate electrode, the negative charges in the solution will be attracted to the gate electrode while the positive charges will accumulate on the surface of channel in the case of Fig. 6(a), i.e, without ISM on the HEMT channel. The formation of EDL on both sides lead to a solution capacitance C_s_ which modulates the transistor dielectric capacitance C_d_. Considering the capacitances are in series, we can define equivalent capacitance as:

4 $$\frac{1}{{C}_{eq}}=\frac{1}{{C}_{1}}+\frac{1}{{C}_{2}}+\mathrm{...}+\frac{1}{{C}_{n}}$$

The electrical impedance in a circuit is defined as

5 $${Z}_{C}=\frac{1}{j\omega C}$$

where *j* and *ω* represent the phase information, respectively.

Thus the voltage drop across the test solution and the transistor dielectric in the sensor without ISM, under the applied gate bias V_g_ are defined as

6 $${\rm{\Delta }}{V}_{S}=\frac{\frac{1}{j\omega {C}_{S}}}{\frac{1}{j\omega {C}_{d}}+\frac{1}{j\omega {C}_{S}}}\times {V}_{g}=\frac{{C}_{d}}{{C}_{d}+{C}_{S}}\times {V}_{g}$$

7 $${\rm{\Delta }}{V}_{d}=\frac{{C}_{s}}{{C}_{d}+{C}_{s}}\times {V}_{g}$$

where Δ*V*_*s*_ and Δ*V*_*ox*_ are the gate voltage drops in solution and in dielectric, respectively.

In the case of Fig. 6(b), i.e., with ISM on the HEMT channel, the EDL is formed on the gate electrode and the surface of the ISM, under applied V_g_. The charges in the ISM will then build up another EDL, causing charge accumulation on the transistor channel, with potential dropping across the transistor dielectric and modulating the drain current. Thus the applied potential drops across the test solution, the ISM and the transistor dielectric, setting up a solution capacitance C_s_, capacitance across ISM C_m_ and capacitance across the dielectric C_d_ respectively. Therefore for an applied gate bias V_g_, we can define the potential drop in the Pb-ISHEMT sensor as:

8 $${\rm{\Delta }}{V}_{S}=\frac{\frac{1}{{C}_{S}}}{\frac{1}{{C}_{S}}+\frac{1}{{C}_{m}}+\frac{1}{{C}_{d}}}\times {V}_{g}=\frac{{C}_{m}{C}_{d}}{{C}_{m}{C}_{d}+{C}_{S}{C}_{d}+{C}_{m}{C}_{S}}\times {V}_{g}$$

9 $${\rm{\Delta }}{V}_{m}=\frac{{C}_{S}{C}_{d}}{{C}_{m}{C}_{d}+{C}_{S}{C}_{d}+{C}_{m}{C}_{S}}\times {V}_{g}$$

10 $${\rm{\Delta }}{V}_{d}=\frac{{C}_{S}{C}_{m}}{{C}_{m}{C}_{d}+{C}_{S}{C}_{d}+{C}_{m}{C}_{S}}\times {V}_{g}$$

where ∆V_s_, ∆V_m_ and ∆V_d_ are the potential drops across the test solution, ISM and the transistor dielectric respectively. In conventional potentiometry, interfacial potential drop which is the potential difference at the interface between solution and membrane is considered. In the high filed-modulated region of our sensor design, because the solution capacitance has become distance-dependent, leading to a fully capacitive model for our sensor, the interfacial potential drop in this region is therefore already included in Cs and Cm, originating from the distribution of the mobile charges in the test solution and the membrane, respectively. Thus, in our sensor model, we do not need to additionally include interfacial potential drop, as in the conventional potentiometry, which has potential drop independent of the distance between two electrodes. The interfacial potential thus usually needs to be included in conventional potentiometry.

The signal of the sensor is the current gain change, which is determined by the voltage drop across the dielectric (∆V_d_). Thus, the sensitivity of our Pb-ISHEMT sensor can be defined as the change in ∆V_d_ over the concentration of lead ions captured in the ISM. Lead ions change ∆V_d_ by modulating the capacitance of the ion selective membrane. Thus sensitivity of Pb-ISHEMT sensor can be expressed as

11 $$\delta =\frac{d{\rm{\Delta }}{V}_{d}}{d\,\mathrm{log}[P{b}^{2+}]}=\frac{d{\rm{\Delta }}{V}_{d}}{d{C}_{m}}\times \frac{d{C}_{m}}{d\,\mathrm{log}[P{b}^{2+}]}$$

Since the concentration of lead ions captured in the ISM is related to the capacitance of ISM, we assume that C_m_ and lead ion concentration can be related with a proportionality as following,

12 $${C}_{m}=k\times \,\mathrm{log}[P{b}^{2+}]$$

where k can either be a constant of proportionality or a function, assuming C_m_ is not influenced by C_s_ or C_d_. Since the effective change in V_g_ results in change in transistor dielectric, ∆V_d_, by combining equations 10–12, we can express sensitivity as

13 $$\begin{array}{rcl}\delta  & = & \frac{{C}_{d}}{{({C}_{m}+{C}_{d})}^{2}+\frac{2({C}_{m}{{C}_{d}}^{2}+{{C}_{m}}^{2}{C}_{d})}{{C}_{S}}+\frac{{{C}_{m}}^{2}{{C}_{d}}^{2}}{{{C}_{S}}^{2}}}{V}_{g}\times k\\  & = & A\times k\end{array}$$

when gap between the gate and the channel increases or when the applied gate voltage decreases, C_s_ decreases. Therefore, for very large gap, C_s_ approaches a minimum value C_min_, as in the saturation region the gain remains constant when the gap keeps increasing, leading to a minimum value A_min_. Therefore,

14 $$\begin{array}{c}{C}_{s}\to {C}_{{\rm{\min }}}\,{\rm{and}}\,A\to {A}_{{\rm{\min }}}\\ {\delta }_{{\rm{\min }}}={A}_{{\rm{\min }}}\times k\end{array}$$

By observing above equation 14, we can see that when C_s_ is very small, as is the case of conventional ISE or ISM FET, sensitivity would be small which approaches ideal Nernst sensitivity, as shown in Fig. 5(c).

On the other hand, for very small gap (or in linear region of device operation), C_s_ becomes infinite, leading to maximum value A_max_. This maximum A indicates that the sensitivity will not keep increasing and become infinite. In stead, there exist a maximum sensitivity when the gap becomes extremely small or Vg becomes extremely large, as shown in equation 15.

15 $$\begin{array}{c}{C}_{s}\to \infty \,{\rm{and}}\,A\to {A}_{{\rm{\max }}}\\ {\delta }_{{\rm{\max }}}={A}_{{\rm{\max }}}\times k=\frac{{C}_{d}}{{({C}_{d}+{C}_{m})}^{2}}{V}_{g}\times k\end{array}$$

Thus with increasing Cs value, as in the design of our Pb-ISHEMT with short gap between the gate electrode and the ISHEMT channel, the sensitivity increases. Therefore, we can obtain sensitivity higher than the ideal Nernst slope and by modulating the sensor bias conditions and the gap between the gate electrode and ISHEMT channel, we can obtain lower detection limits, comparable to the laboratory equipments such as ICP-MS.

## Conclusion

In this research, we have demonstrated a whole new methodology for ion-selective membrane coated FET sensors, which can easily create much larger sensitivity surpassing the limit of ideal Nernst sensitivity. The increased sensitivity tremendously improves the detection limit for several orders of magnitude of lead ions. Lead ion selective high electron mobility transistor (Pb-ISHEMT) was used to prove this methodology. Systematical investigation was done by measuring different design of the sensor and gate bias, indicating that in a strong field environment, the sensitivity can surpass the ideal Nernst equation. It suggests that with our design, the sensitivity (−36 mV/log [Pb^2+^]) can overcome the restriction of the ideal Nernst slope (−29.58 mV/log[Pb^2+^]). Theoretical study in the sensitivity consistently agrees with the experimental finding and predicts an existing maximum sensitivity. Usually, a traditional Pb ISE has a detection limit of 10^−7^ M. For our sensor, the detection limit can be as low as 10^−10^ M and by modulating bias and device configuration, even lower detection limits can be achieved. This result is comparable to that of ICP-MS which also has detection limit around 10^−10^ M to 10^−11^ M. In contrast, other laboratory based equipments which are quite bulky and expensive, only have a detection limit of 10^−8^ M. Finally, the convenience, low cost and ease of fabrication by batch production enable our sensor with a competitive market value. The sensing mechanism developed in this paper can be applied to all ion selective membrane FETs. By incorporating this design, we can develop a reliable commercial sensor for rapid screening of heavy metal ion contamination in natural ecosystems, water dispensers, foods and beverages.

## Methods

### Fabrication of AlGaN/GaN High electron mobility transistors (HEMTs)

Fabrication of the AlGaN/GaN HEMT starts with the growth of AlGaN/GaN epi-layer on a silicon substrate by the metal-organic chemical vapor deposition (MOCVD) system. The double layer construction includes a 3 µm thick GaN layer followed by a 150 Å thick AlGaN layer. As the AlGaN layer provides high polarization, 2-DEG (two-dimensional electron gas) is generated at the junction between GaN and AlGaN layer. The device active area is defined by mesa etch process using inductively coupled plasma (ICP) etching with Cl_2_/BCl_3_ gases under ICP power of 300 W and RF bias of 120 W at 2 MHz. Source-drain metals are then deposited using electron beam (e-beam) evaporator in consecutive layers of 200 Å of Ti, 400 Å of Al, 800 Å of Ni and 1000 Å of Au. Ohmic contacts are formed by performing two-step rapid thermal annealing (RTA) process at 200 °C for 25 seconds and gradually increasing to 850 °C for 40 seconds. Metal interconnects and in-plane reference electrodes are deposited using evaporator with 200 Å Ti and 1000 Å Au. Finally, the entire device is passivated using SU-8 photoresist and channel and gate electrode areas are patterned using photolithography to yield openings of 10 × 60 µm^2^ and 100 × 120 µm^2^ respectively.

### ISM preparation and immobilization

The lead ion selective membrane consists of lead 1 wt % ionophore IV, 0.35 wt % Potassium tetrakis(4-chloropheny)borate as added anion, 65.65 wt % 2-Nitrophenyl octyl ether (2NOE) as plasticizer, and 33 wt % Poly(vinyl chloride) high molecular weight (PVC) as a substrate. The total amount of ISM components of 300 mg is dissolved in 3 ml of tetrahydrofuran (THF), a versatile organic solvent. ISM will then be stored on amber glass bottle sealed using para-film.

To fabricate a Pb-ISHEMT chip, 0.5 μl of ISM mixture is drop cast on the SU-8-passivated HEMT chip to cover the channel open area alone. The opening on the gate electrode is kept clean during the process. The device is incubated in a well-ventilated environment in room temperature for at least 24 hours for the evaporation of organic solvent. In this structure, ISM on the channel acts as the Pb^2+^ ion receptor capturing the Pb^2+^ ions in the test solution, and the in-plane gate reference electrode serves to modulate the transistor drain current via EDL formation.

### Pb^2+^ ion samples

Target Pb^2+^ ions are prepared in 0.02X PBS (Phosphate buffered saline) with de-ionized (DI) water. The water sources are calibrated using gold standard equipment ICP-MS to determine the concentration of Pb^2+^ to be less than 10^−13^ M. The pH of PBS buffer is maintained close to neutral for optimal ISM activity.

### Sensor regeneration

After testing with Pb^2+^ ion containing solution, the sensor is washed and incubated in pure 0.02X PBS solution for 1–3 hours to regenerate the ISM for further testing. The sensor regeneration can be validated through electrical testing. The sensor baseline is restored after regeneration.

### Sensor measurement

Agilent B1530/B1500A semiconductor parameter analyzer is used to measure the transistor characteristics. A short duration gate pulse with a width of 2 ms and amplitude of 1 V is applied as the gate bias to the reference electrode. A steady DC bias of 2 V is applied as the drain-source voltage during device operation.

## Electronic supplementary material

Supplementary information
