# Supplementary material for: High-field modulated ion-selective field-effect-transistor (FET) sensors with sensitivity higher than the ideal Nernst sensitivity
Source: Sci Rep. 2018 May 29;8:8300. doi: 10.1038/s41598-018-26792-9 (PMC5974191; doi:10.1038/s41598-018-26792-9)
Supplement: Supplementary file 1 — Supplementary information [file 41598_2018_26792_MOESM1_ESM.doc]

**High-field modulated ion-selective field-effect-transistor (FET) sensors with sensitivity higher than the ideal Nernst sensitivity**

Yi-Ting Chen^1^, Indu Sarangadharan^1^, Revathi Sukesan^1^, Ching-Yen Hseih^2^, Geng-Yen Lee^3^, Jen-Inn Chyi^3^ and Yu-Lin Wang*^1, 2^

1. Institute of Nanoengineering and Microsystems, National Tsing Hua University, Hsinchu, 300, Taiwan, R.O.C.

2. Department of Power Mechanical Engineering, National Tsing Hua University, Hsinchu 300, Taiwan, R.O.C.

3. Department of Electrical engineering, National Central University, Jhongli City, Taoyuan County 320, Taiwan, R.O.C.

**Supplementary Information**

**Pb-ISHEMT sensing characteristics**

To evaluate the transfer characteristics of Pb-ISHEMT sensor, DC gate sweep voltage is applied to the gate electrode, at a drain voltage of 2 V. The transfer curve is depicted in Figure S1, which demonstrates the sensor response in different concentrations of lead ion in 0.02X PBS. With increasing lead ion concentration, the drain current decreases. However, throughout our study, Pb-ISHEMT is operated under a short duration gate pulse, to avoid the variations arising from thermal effects due to continued DC operation and to prevent redox currents. The output drain current characteristics (I_d_ versus time) thus obtained are shown in Figure S2. With increasing lead ion concentration, the drain current of ISHEMT decreases. A 2 ms pulse voltage of amplitude 1 V is applied as the gate bias and a steady DC voltage of 2 V is applied as the drain bias. Since absolute drain current is prone to variations, we chose the difference in drain current with and without applied Vg, called current gain or simply ‘gain’, as our sensor index of measurement. The gain values thus calculated are used throughout in the study to elucidate the sensing characteristics.

Figure S1 Transfer characteristics of Pb-ISHEMT sensor.


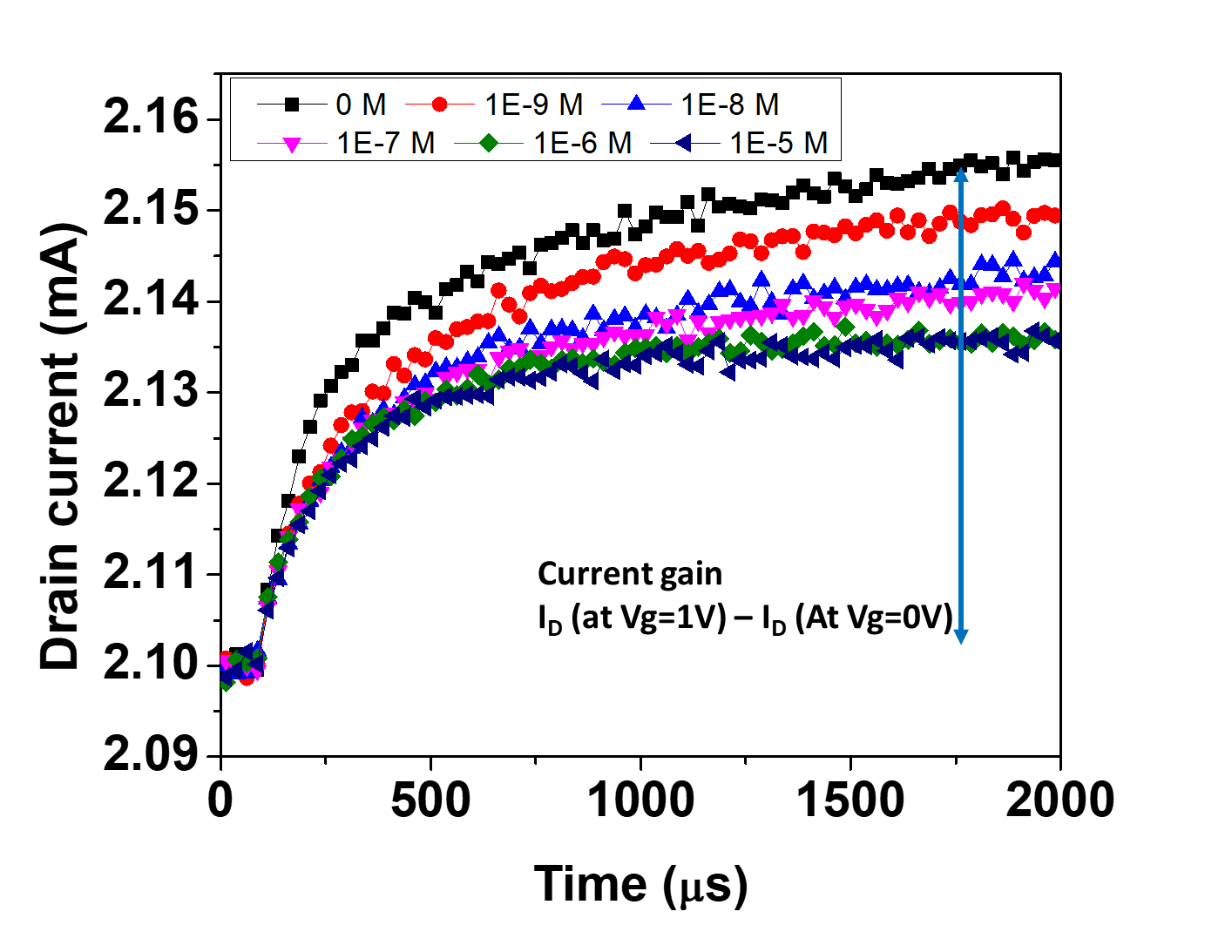


Figure S2 Drain current versus time graph for lead ion detection using ISHEMT sensor.
